# Impact of the Bayesian penalized likelihood algorithm (Q.Clear®) in comparison with the OSEM reconstruction on low contrast PET hypoxic images

**DOI:** 10.1186/s40658-020-00300-3

**Published:** 2020-05-12

**Authors:** Edgar Texte, Pierrick Gouel, Sébastien Thureau, Justine Lequesne, Bertrand Barres, Agathe Edet-Sanson, Pierre Decazes, Pierre Vera, Sébastien Hapdey

**Affiliations:** 1 Nuclear Medicine Department, Henri Becquerel Cancer Center, Rouen, France; 2 QuantIF-LITIS EA4108, Rouen University Hospital, Rouen, France; 3 Radiotherapy Department, Henri Becquerel Cancer Center, Rouen, France; 4 Clinical Research Department, Henri Becquerel Cancer Center, Rouen, France; 5 Nuclear Medicine Department, Jean Perrin Cancer Center, Clermont-Ferrand, France

**Keywords:** PET/CT, Hypoxia, NSCLC, BPL reconstruction

## Abstract

**Purpose:**

To determine the impact of the Bayesian penalized likelihood (BPL) reconstruction algorithm in comparison to OSEM on hypoxia PET/CT images of NSCLC using ^18^F-MIZO and ^18^F-FAZA.

**Materials and methods:**

Images of low-contrasted (SBR = 3) micro-spheres of Jaszczak phantom were acquired. Twenty patients with lung neoplasia were included. Each patient benefitted from ^18^F-MISO and/or ^18^F-FAZA PET/CT exams, reconstructed with OSEM and BPL. Lesion was considered as hypoxic if the lesion SUV_max_ > 1.4. A blind evaluation of lesion detectability and image quality was performed on a set of 78 randomized BPL and OSEM images by 10 nuclear physicians. SUV_max_, SUV_mean,_ and hypoxic volumes using 3 thresholding approaches were measured and compared for each reconstruction.

**Results:**

The phantom and patient datasets showed a significant increase of quantitative parameters using BPL compared to OSEM but had no impact on detectability. The optimal beta parameter determined by the phantom analysis was *β*350. Regarding patient data, there was no clear trend of image quality improvement using BPL. There was no correlation between SUV_max_ increase with BPL and either SUV or hypoxic volume from the initial OSEM reconstruction. Hypoxic volume obtained by a SUV > 1.4 thresholding was not impacted by the BPL reconstruction parameter.

**Conclusion:**

BPL allows a significant increase in quantitative parameters and contrast without significantly improving the lesion detectability or image quality. The variation in hypoxic volume by BPL depends on the method used but SUV > 1.4 thresholding seems to be the more robust method, not impacted by the reconstruction method (BPL or OSEM).

**Trial registration:**

ClinicalTrials.gov, NCT02490696. Registered 1 June 2015

## Key points

Question: Is there any interest to use the BPL reconstruction on low-contrasted PET images of hypoxia in terms of lesion detectability, quantification, and delineation.

Pertinent findings: BPL algorithm improves quantitative measurements and contrast but does not improve the lesion detectability on low-contrasted images compared to OSEM algorithm. The variation in hypoxic volume by BPL depends on the segmentation method used but SUV > 1.4 thresholding appears to be the more robust method, not impacted by the reconstruction method (BPL or OSEM), nor the BPL parameters.

Implications for patient care: More accurate PET hypoxic images can be obtained using BPL reconstruction and used with more confidence in the patient radiation treatment planning.

## Introduction

^18^F-Fluorodeoxyglyucose (^18^F-FDG) PET/CT is a commonly used imaging modality to help in diagnosing and stratifying diseases with various indications in oncology, cardiology, infectiology, or rheumatology.

In oncology, several studies have shown the interest to use metabolic information from PET/CT to optimize radiotherapy delineation [1] with ^18^F-FDG. Some studies tried to show the interest to intensify radiotherapy on hypoxic volume of lung cancer to increase radiotherapy efficiency [2, 3]. More recently, Vera et al. underlined the strong correlation between ^18^F-MISO uptake and poor prognosis the improvement of survival for patients treated with a radiotherapy boost on the hypoxic volume of non-small cell lung carcinoma (NSCLC) [4]. ^18^F-MISO ([1H-1-(3-[18F]fluoro-2-hydroxypropyl)-2-nitroimidazole) and ^18^F-FAZA ([18F]fluoroazomycin arabinoside) are radiopharmaceuticals revealing the hypoxic areas of tumoral disease. Unfortunately, ^18^F^-^MISO and ^18^F-FAZA show a low signal to background ratio (SBR) leading to difficulties in visualization, segmentation, and quantification of the lesion and the subsequent irradiation volume determination.

Reconstruction of PET raw data is based on iterative methods, the most commonly used being the ordered subset expectation maximization (OSEM). Those approaches require setting up a number of subsets and iterations to reconstruct the image. Theoretically, the higher the number of subsets or iterations, the closer to the expected reconstructed image. However, the increase of subsets and iterations generates noise that worsens image quality and causes misinterpretations, quantification, and potentially segmentation errors.

The Bayesian penalized likelihood (BPL) PET reconstruction algorithm (Q.Clear—GE Healthcare, Milwaukee, WI) is an algorithm, newly proposed on the General Electric PET devices, based on a point spread function (PSF) modeling and a penalizing function reducing noise between each iteration (driven by a so-called *β*). This algorithm allows the use of high number of subset and iterations, improving contrast while preventing any noise increase [5, 6].

Most of the studies about the BPL algorithm showed the benefit with ^18^F-FDG PET/CT on various types of lesions [7–12]. To our knowledge, there is no publication assessing the usefulness of BPL on small lesions with low signal to background ratio (SBR).

Thus, the purpose of this study is to evaluate the contribution of the BPL reconstruction algorithm in low contrast PET/CT images, in terms of quantification, detectability, and image quality compared to OSEM reconstruction. The evaluation was first performed on phantom images, and then on images from patients with pulmonary neoplasia who benefitted from PET/CT examinations with hypoxia tracers (^18^F-MIZO and ^18^F-FAZA).

## Materials and methods

### Phantom

A Jaszczak phantom with the 6 fillable micro-spheres (diameters/volumes of 5.94 mm/31 μL, 6.95 mm/63 μL, 8.23 mm/125 μL, 9.86 mm/250 μL, 11.89 mm /500 μL, 14.43 mm/1000 μL) was scanned on our LYSO-based Discovery 710 PET/CT system (GE Healthcare). First, 26.4 MBq of ^18^FDG was injected in the phantom tank filled with 2 L of water. We withdraw 2 mL from this mixture to fill the 6 micro-spheres. We finally filled the rest of the phantom tank with water up to its maximum capacity (6.2 L) which gave a contrast ratio between spheres and background of 3.1:1.

The phantom was centered on the center of the field-of-view and a 20-min list-mod acquisition over one bed position was performed, allowing the reconstruction of different acquisition times (2, 5, 10, 15, and 20 min). The raw data were reconstructed according to the routinely used OSEM protocol (2 iterations and 24 subsets with a 6.4-mm Gaussian filter, including correction of time of flight, attenuation, scatter, and incorporating the point spread function—SharpIR) and with the BPL algorithm (*β* parameter set up to 300, 350, 400, 500, and 600).

### Patients

We used PET/CT datasets of patient included in an ongoing study (RTEP6, NCT02490696) in our center that aim to compare ^18^F-MISO and ^18^F-FAZA in NSCLC where each patient benefit from ^18^F-FDG, ^18^F-MISO, and ^18^F-FAZA PET/CT scans before their surgery.

^18^F-MISO and ^18^F-FAZA PET/CT scans were acquired 180 min after the injection of 4 MBq/kg of radiopharmaceutical, with an acquisition time of 4 min/bed position. CT scan was set up to 80 mAS and 100 kV, with an intensity modulation system, yielding a mean DLP value of 160.9 ± 44 mGycm. As for the phantom, OSEM reconstructions were performed using 2 iterations and 24 subsets with 6.4 mm Gaussian post-filter which corresponds to our clinical parameters. We decided to use the BPL algorithm with *β* value of 300, 350, and 400 based on the review of the literature for which the optimal *β* was frequently chosen between 300 and 400 [5, 7] and considering the results obtained on the phantom data. All patients gave their written informed consent.

### Image analysis

#### Quantification

On the phantom data, spherical volumes of interest (VOIs) were manually drawn on the 20-min BPL350-reconstructed PET images, on each visible sphere and on the background (1-cm^3^ spherical VOI) to measure quantitative parameters (SUV_max_ and SUV_mean_). Each VOI was then perfectly cloned on every sequence (all reconstructions and all acquisition times) to get the measurements on the exact same location and prevent any intra-operator variability.

Then, we determined the contrast recovery coefficient (CRC) and background variability (BV) by using a formula previously proposed [5]:

$$ \mathrm{CRC}=\frac{\frac{{\mathrm{SUV}}_{\mathrm{mean}}\mathrm{sphere}}{{\mathrm{SUV}}_{\mathrm{mean}}\mathrm{background}}}{\frac{\mathrm{Activity}\kern0.17em \mathrm{in}\mathrm{jected}\kern0.17em \mathrm{in}\kern0.17em \mathrm{sphere}}{\mathrm{Background}\kern0.17em \mathrm{Actity}}}\mathrm{and}\;\mathrm{BV}=\frac{\mathrm{SD}\;\mathrm{background}}{{\mathrm{SUV}}_{\mathrm{mean}}\mathrm{background}}\times 100 $$

The percentage difference of SUV_max_, SUV_mean_, SUV_peak_, CRC, and BV was also calculated (ΔSUV_max_, ΔSUV_mean_, ΔSUV_peak_, ΔCRC, and ΔBV, respectively).

On the patient images, for each reconstruction, the lesion was considered as hypoxic if the lesion SUV_max_ was superior to 1.4, as it was proposed by Thureau et al. [13].

Hypoxic volumes were then delineated using three different methods:

1. A threshold expressed as 1.5-fold the mediastinum SUV_max_ (Th_1.5Med_) [13]
2. A fixed threshold based on SUV values > 1.4 (Th_1.4_) [3, 13]
3. A 60% thresholding method (Th_60%_), containing all voxels with a value superior or equal to 60% of the SUV_max_ value.

We studied the correlation between SUV change (ΔSUV) with BPL as a function of the initial SUV using a Bland-Altman analysis and the correlation with the initial volume with a scatter plot.

In complement to the quantitative analysis, we realized a blind evaluation of detectability and image quality of lung lesions. To that end, we used a random subset of non-CT-fused 2D OSEM and BPL PET images showing each lesion twice (OSEM and BPL) but not consecutively allowing a paired statistical analysis (for BPL, b350 was chosen as a trade-off between a CRC increase and noise limitation). All these images contained the whole lungs and the observers were informed of the presence of a lesion on each slice. Ten senior nuclear medicine physicians from two centers evaluated (1) the detectability of a lesion asking if a lesion was considered visible (binary answer) and (2) the overall quality of the image, considering contrast, SNR, and background noise level. The quality was ranked with a 5-point Likert-like scale (1, uninterpretable; 2, poor; 3, correct; 4, good; 5, excellent). All the images were evaluated in one session and there was no waiting period between different images.

For the detectability analysis, we calculated for each PET/CT exam and reconstruction, the total number of lesions detected by the 10 observers.

For the image quality analysis, we calculated the total score attributed by each observer (with a maximum score of 5/5 per image). Then, we measured the total quality score for each reconstruction and for each observer independently.

### Statistical analysis

We compared the phantom quantitative data (ΔSUV_max_, ΔSUV_mean_, and ΔCRC) and hypoxic volumes using a Wilcoxon paired ranked-sum test. For the clinical analysis, we compared OSEM and BPL quantitative parameters (SUV_max_, SUV_mean_) using a Student paired *t* test. *p* values less than 0.05 were considered statistically significant.

Detectability results were compared using a Cochran’s Q test. The comparison of lesion detectability between OSEM and BPL reconstructions was evaluated by calculating the kappa coefficients for each observer. The results of the image quality comparison between OSEM and BPL reconstructions were represented in contingence table and evaluated by calculating weighted kappa coefficients for each observer.

All graphs and plots were realized with MedCalc 13.1.2.0 and Excel 2010.

## Results

### Phantom evaluation

**Fig. 1:**
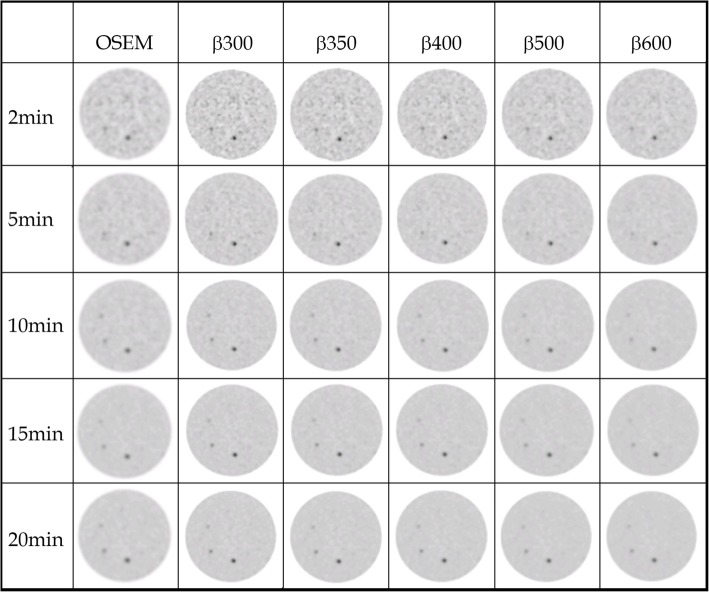
Results of Jaszczak phantom acquisition for each time and each reconstruction

Figure 1 presents the images of the phantom, for the 6 reconstructions and for the 5 acquisition times. At 2 min and 5 min acquisition time, only the two biggest spheres can be detected on all reconstructions. Acquisition of 10 and 15 min per bed allows detecting three of the six spheres, and 20-min acquisition permits to slightly see the fourth one.

**Fig. 2:**
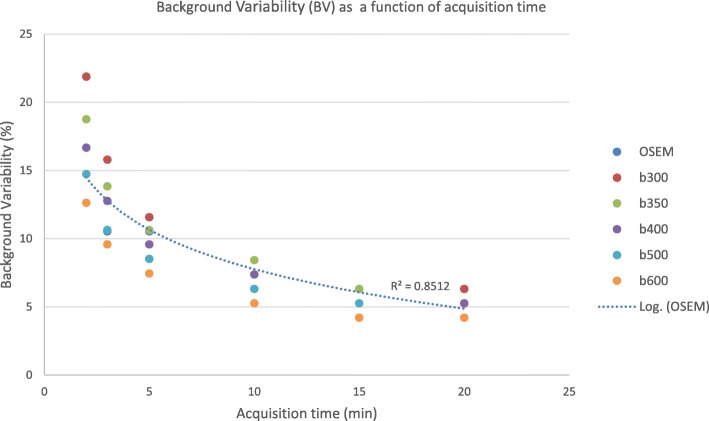
Background variability as a function of the acquisition time and reconstruction method: OSEM and BPL with a beta parameter from 300 to 600. The dashed line represents the logarithmic fitting of OSEM values

Figure 2 shows the BV as the function of time, at 2, 3, 5, 10, 15, and 20 min. A comparable background noise level is observed between OSEM and BPL with *β*350–400 and starts to be lower in BPL than OSEM at *β*500. Noise level at *β*300 is higher than OSEM at all time per bed.

Table 1 gives the quantitative modification between the OSEM and the BPL reconstructions. There was a statistically significant increase of SUV_max_ and SUV_mean_ on all visible spheres and regardless of acquisition time, except with BPL with *β*600. For instance, on the 2 min acquisition, the SUV_max_ increase ranged from + 11.6% at *β*500 (*p* = 0.012) to + 37.2% at *β*300 (*p* < 0.001).

**Table 1:** Mean values of ΔSUV_max_, ΔSUV_mean_, and ΔCRC (contrast recovery coefficient) averaged over the 5 acquisition times and all detected spheres (*n* = 14), between BPL with different *β* parameter and OSEM reconstructions

	*β*300	*β*350	*β*400	*β*500	*β*600
ΔSUV_max_	+37.18%	+28.52%	+21.66%	+11.55%	+3.25%
*p value*	*0.0001*	*0.0002*	*0.0012*	*0.13*	*0.38*
ΔSUV_mean_	+27.62%	+22.18%	+17.57%	+9.62%	+2.93%
*p value*	*0.0001*	*0.0002*	*0.0017*	*0.22*	*0.45*
ΔCRC	+30.85%	+27.89%	+21.22%	+12.65%	+5.71%
*p value*	*0.0002*	*0.0004*	*0.0024*	*0.20*	*0.34*

**Fig. 3:**
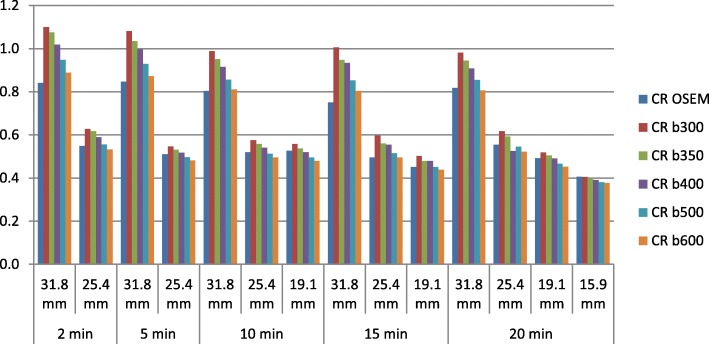
Contrast recovery (CRC) as a function of sphere diameter and reconstruction method (OSEM and BPL with a beta of 300 to 600)

Figure 3 plots the contrast recovery for each visible sphere as a function of the acquisition time, and Table 1, the improvement between BPL with respect to OSEM reconstruction. Figure 3 illustrates that BPL has a clear benefit on contrast recovery with all *β* parameters on the largest sphere whatever the acquisition time. For the second and third spheres, BPL seemed to give higher contrast recovery at *β*300, *β*350, and *β*400. On the other hand, there was no significant gain of contrast recovery at BPL for *β*500 and *β*600.

Considering these results, we chose to use *β*350 for the BPL reconstruction, as the best compromise between noise control and quantitative contrast recovery, for the detectability and image quality analysis of the clinical images.

### Patients

Patient characteristics are summarized in Table 2.

**Table 2:** Patient characteristics

Patient	*n* = 20 (%)
Sex	
Men	18 (90)
Women	2 (10)
Age (mean)	65 (50–83)
ECOG	
0	11 (55)
1	8 (40)
2	1 (5)
3	0
4	0
Histology	
SCC	10 (50)
Adenocarcinoma	9 (45)
Other	1 (5)

*ECOG* Eastern Cooperative Oncology Group Performance status, *SCC* squamous cell carcinoma

We analyzed data from 20 patients (18 males/2 females) included in the RTEP6 study between 2016 and 2018 which aims to compare 18F-MISO and 18F-FAZA PET/CT scans in patients with a suspicion of lung neoplasia. Eighteen patients benefitted from both ^18^F-MISO and ^18^F-FAZA PET/CT scans, 1 patient had only one ^18^F-MISO PET/CT and another one had only one ^18^F-FAZA PET/CT. One patient had no cancerous cell found after surgery and the pathological analysis concluded to an organized infectious pneumopathy. In total, 38 PET/CT scans of 20 different lesions were analyzed.

### Lesion detectability and image quality

Table 3 gives the number of lesions detected by the 10 observers. For all observers, the lesions were detected in 329/380 cases with the OSEM reconstruction and in 322/380 cases with the BPL reconstruction. Lesions were detected by the 10 observers in 24/38 cases (63.2%) with OSEM and 25/38 cases (65.8%) with BPL. The lesion detectability was increased with BPL in 5 cases and lowered in 12 cases. There was a significant difference between OSEM and BPL for only one observer on Cochran’s *Q* test showing a better detectability with OSEM than BPL (*p* = 0.046). Cochran’s *Q* test was not realizable for observer 10 because of exact same results for all OSEM and BPL images. Kappa values for the ten observers were ranged from 0.47 to 1 traducing the major impact that reconstruction can have on lesion detectability for a same patient.

**Table 3:** Number of lesions detected by observer and by reconstruction

DS	OBS1	OBS2	OBS3	OBS4	OBS5	OBS6	OBS7	OBS8	OBS9	OBS10
OSEM	33	**36**	**35**	**34**	28	31	**33**	**29**	**33**	37
BPL	33	34	32	30	**31**	**33**	32	28	32	37
*Cochran’s p value*	*1*	*0.157*	*0.083*	*0.046*	*0.18*	*0.317*	*0.655*	*0.705*	*0.317*	*x*
kappa	*0.77*	*0.69*	*0.68*	*0.66*	*0.64*	*0.62*	*0.47*	*0.51*	*0.90*	*1*

*OSEM* ordered subset expectation maximization, *BPL* Bayesian penalized likelihood algorithm

Tables 4 and 5 present the distribution of the quality score for all observers. On the 380 comparisons of image quality between BPL and OSEM, 108 were in benefit of BPL (28.4%), 103 were in benefit of OSEM (27.1%) and 169 cases showed no change of quality score. The weighted kappa values ranged from 0.092 to 0.612. Quality scores of BPL images were higher for 7 observers than with OSEM.

**Table 4:** Concordance table of quality score of all observers

		BPL				
		1	2	3	4	5
OSEM	1	7	8	0	0	0
	2	14	64	33	10	1
	3	2	47	54	36	10
	4	0	6	30	35	10
	5	1	0	0	3	9

*OSEM* ordered subset expectation maximization, *BPL* Bayesian penalized likelihood algorithm

**Table 5:** Quality score (QS) by observer and by reconstruction method

QS	OBS1	OBS2	OBS3	OBS4	OBS5	OBS6	OBS7	OBS8	OBS9	OBS10
OSEM	104	**123**	**101**	**109**	95	106	110	101	110	136
BPL	**106**	96	96	84	**101**	**116**	**147**	**102**	**120**	**140**
*Weighted kappa*	*0.615*	*0.176*	*0.306*	*0.092*	*0.409*	*0.533*	*0.150*	*0.353*	*0.406*	*0.612*

*OSEM* ordered subset expectation maximization, *BPL* Bayesian penalized likelihood algorithm

**Fig. 4:**
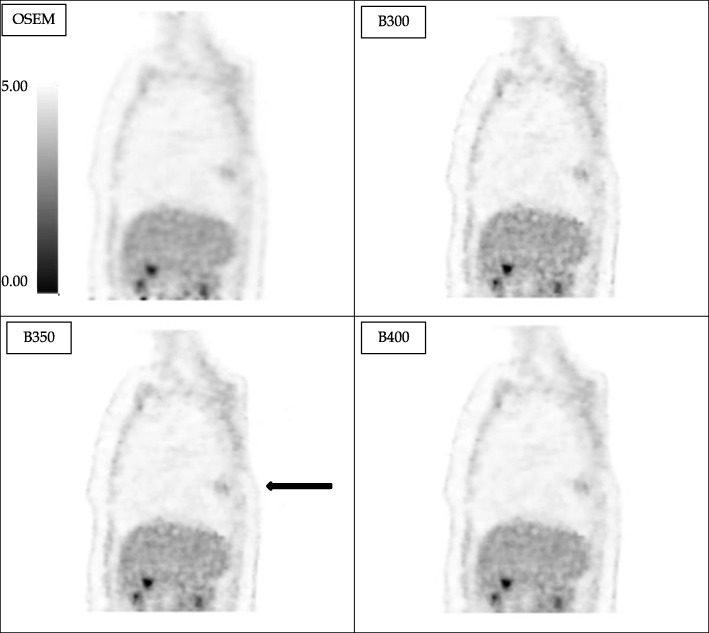
Example of 18F-FAZA thoracic PET images reconstructed with OSEM or BPL (with beta values of 300, 350, 400, and 500; voxel size of 2.73 × 2.73 × 3.27 mm and acquisition time of 4 min/bed position). In that case, BPL (with beta = 350) permits a better detectability of the pulmonary opacity (black arrow) compared to OSEM with a score of 10/10 versus 8/10

With BPL, images were less considered as “correct” but mainly as “good” or “excellent.” Nevertheless, there were more cases of “uninterpretable” compared to OSEM. Figure 4 is a concordance table presenting the quality scores obtained for each comparison of a same image reconstructed with OSEM and BPL (38 pairs of images reviewed by the 10 observers) showing that, in the 108 cases where BPL was preferred, 87 cases showed a gain of 1 point (for example, from 3, correct, to 4, good), 20 cases showed a gain of 2 points and 1 case showed a gain of 3 points. There was no modification of the quality score in 169/380 cases (44.5%). One hundred and three comparisons were in favor of OSEM with a loss of 1 point in 94 cases, 2 points in 8 cases, and 4 points in 1 case.

Table 6 summarizes the results of the quantitative analysis on clinical images. As for our phantom study, the BPL reconstruction leads to a significant higher SUV_max_, SUV_mean_, and SUV_peak_ compared to OSEM on each reconstruction, *β*300 presenting the largest increase. SUV_max_ increases ranged from 10.4 up to 21.5% depending of *β*.

**Table 6:** Mean values (± SD) of quantitative parameters as a function of the reconstruction method (OSEM, BPL b300, b350, and b400) and BPL values variations compared to OSEM reconstruction

	*β*300	*β*350	*β*400	OSEM
mean SUV_max_	3.04 (± 1.48)	2.87 (± 1.33)	2.77 (± 1.30)	2.50 (± 1.12)
*% of variation compared to OSEM*	*+21.5%*	*+14.6%*	*+10.4%*	
*p value*	*< 0.001*	*< 0.001*	*< 0.001*	
mean SUV_mean_	1.62 (± 0.67)	1.57 (± 0.66)	1.55 (± 0.64)	1.47 (± 0.60)
*% of variation compared to OSEM*	*+10.1%*	*+6.9%*	*+5.2%*	
*p value*	*< 0.001*	*< 0.001*	*< 0.001*	
mean SUV_peak_	2.20 (± 1.03)	2.17 (± 1.01)	2.15 (± 1.01)	2.07 (± 0.94)
*% of variation compared to OSEM*	*+6.5%*	*+4.9%*	*+4%*	
*p value*	*< 0.001*	*< 0.001*	*< 0.001*	

**Fig. 5:**
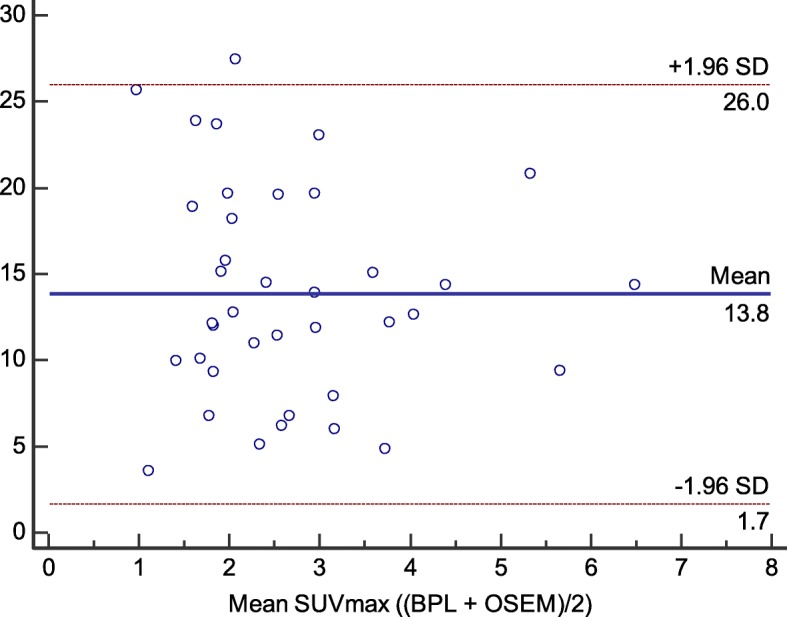
Bland-Altman plot of SUV_max_ increase (%) as a function of initial SUV_max_ on the OSEM reconstruction using BPL with *β*350

On the Bland-Altman plot represented on Fig. 5, we see the absence of correlation between SUV_max_ increase with the BPL reconstruction and initial SUV_max_ on OSEM reconstruction.

Due to the quantification increase with BPL, there was one more lesion considered as “hypoxic” with BPL (36/38 at b350) than OSEM (35/38 lesions) considering our decision criteria (SUV_max_ > 1.4).

With the OSEM reconstruction, hypoxic volume could be measured on all the 35 hypoxic lesions with Th_60%_ and Th_1.4_ segmentation methods but only on 12 lesions with the Th_1.5Med_ method. BPL allowed to measure 15 hypoxic volumes with the Th_1.5Med_ segmentation method with *β* = 350 and *β* = 400 and 16 volumes with *β* = 300.

Table 7 gives the hypoxic volume measurements, considering the 3 segmentation methods. On these 35 hypoxic lesions, the Th_60%_ method showed a significant reduction of hypoxic volume between OSEM and BPL and with each *β* parameter (*p* < 0.001). The Th_1.4_ method showed a stable hypoxic volume between OSEM and BPL, whatever the *β* value considered (*β* = 300, 350, or 400). The Th_1.5Med_ segmentation method trends to give lower hypoxic volumes than OSEM but with no significant difference.

**Table 7:** Mean (± SD) value of metabolic volume (expressed in cm^3^) as a function of the reconstruction method (OSEM, BPL b300, b350, and b400) and segmentation method

Method	*β*300	*β*350	*β*400	OSEM
Th_60%_	5.56 (± 2.64)	6.17 (± 3.20)	6.36 (± 3.40)	10.13 (± 5.48)
*p value*	*< 0.001*	*< 0.001*	*< 0.001*	
Th_1.4_	30.43 (± 11.64)	*30.69* (± 11.87)	*29.28* (± 11.39)	29.07 (± 11.38)
*p value*	*0.23*	*0.38*	*0.96*	
Th_1.5Med_	3.24 (± 2.87)	4.13 (± 3.86)	4.36(± 4.01)	4.40 (± 4.40)
*p value*	*0.52*	*0.17*	*0.42*	

**Fig. 6:**
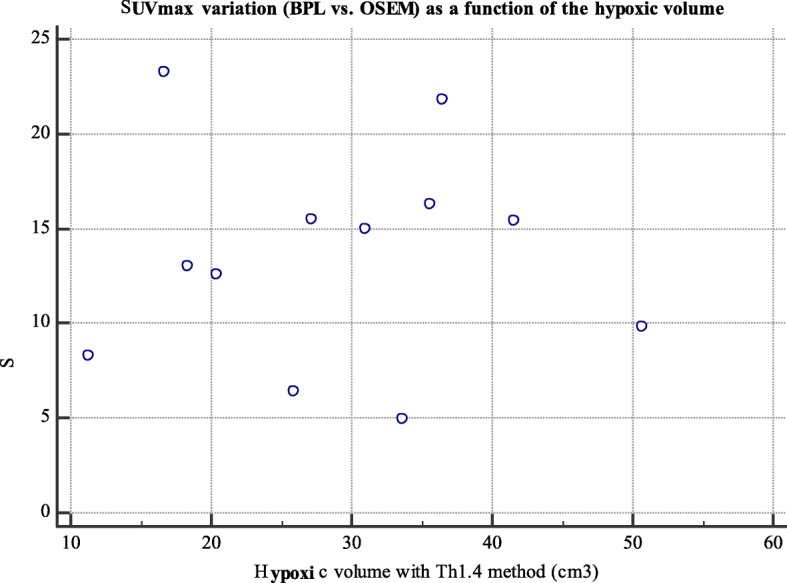
Scatter plot of SUV_max_ increase as a function of initial metabolic volume on the OSEM reconstruction (only considering hypoxic lesions *n* = 12)

Figure 6 shows that there is no correlation between the variation in SUV_max_ when using BPL vs. OSEM and the hypoxic volume determined by the Th_1.4_ segmentation method.

## Discussion

This study aimed to evaluate the benefit of the BPL reconstruction algorithm on PET/CT images of hypoxia presenting low-contrasted lesions. Our results suggest that the BPL algorithm clearly increase quantitative parameters and contrast on PET/CT reconstruction which is concordant with all other papers studying this reconstruction algorithm [5, 8, 10, 14].

Our optimal *β* parameter was selected according to our phantom analysis showing that *β*350 had a contrast recovery coefficient close to 1 (using SUV_mean_ values) and a noise level comparable to OSEM, unlike *β*300. This is in line with a study realized on a LYSO-based PET/CT scanner by Teoh et al., which proposed to use a *β* value of 400 [5], and studies realized on a BGO-based PET/CT scanner by Vallot et al. and Reynés-Llompart et al. which proposed an optimal *β* of 400 [15] and 350 [16], respectively. A more recent study realized by Caribé et al. suggested that the optimal beta value depends of the contrast and the lesion’s size but is optimal for maximizing CR and noise level for *β* values ranged between 300 and 400 based on a NEMA phantom experiment with bigger spheres [12]. Another study from Otani et al. evaluated BPL versus OSEM on FDG PET/CT images of lung tumors [17] and proposed a higher optimal *β* value of 500. Indeed, they chose to improve the image quality (lowering the noise level) while preserving the same lesion quantification. At the opposite, we decided to maintain the same noise level than the OSEM reconstruction, but improving the image quantitation to try to improve the lesion detectability. Although their PET/CT device usually used a BGO-based system, this is in line with our results in Fig. 1 and Table 1, where a *β* parameter of 600 (and even 500) induced a noise reduction but a comparable SUV quantitation than with OSEM reconstruction.

Figures 5 and 6 showed that there was no correlation between the SUV_max_ increase with BPL and the initial SUV parameters or metabolic volumes on initial OSEM image. BPL does not benefit more to low contrasted PET/CT images or small metabolic lesions which is not concordant with Teoh et al. study regarding small pulmonary lung nodule [10]. This difference can be easily explained since the lack of benefit on small spheres on our phantom study is due to low activity injected in spheres while pulmonary nodules studied in Teoh’s study had higher uptakes even on OSEM reconstruction. BPL can accentuate the lesion contrast and give a cleaner image, but if there is no signal present in the ground truth there is no chance that the BPL algorithm will produce it.

We categorized lesion as hypoxic or not if at least one voxel signal is superior to 1.4 as it is the only validated method to our knowledge and was used in a previous clinical trial [3].

BPL reconstructions showed a significant decrease of metabolic volume compared to OSEM using a percentage thresholding method (Th_60%_) because the BPL algorithm does not enhance the uptake globally but increase hotspots by restoring point spread function (PSF). These results are concordant with another study showing the reduction of metabolic volume with BPL [15]. Th_1.4_ and Th_1.5Med_ are two segmentation methods that have been proven to be better to evaluate metabolic volume on low contrast PET/CT [3, 13]. With the Th_1.5Med_ method, the metabolic volume tends to be lower, and in some cases, non-measurable. Our results suggest that the Th_1.4_ segmentation method is not impacted by BPL compared to OSEM reconstruction. The BPL reconstruction may lead to important changes in hypoxic tumor volume determined on hypoxia PET and its impact to radiotherapy have to be evaluated. Unfortunately, this means knowing the lesion ground truth, which is complicated in practice.

Background variability increased with the BPL reconstruction which is concordant with Vallot’s study showing a significant increase of hepatic SNR which is mainly relying on an increase of hepatic SUV_mean_ [15]. Indeed, in Fig. 2, background variability values are higher for 2, 10, and 15 min acquisition time which is confirmed on Figs. 1 and 4, where BPL images at *β*350 appear more noisy than OSEM images. But *β*350 was chosen as a trade-off between contrast improvement while remaining at the same noise level as OSEM.

Our detectability analysis did not allow us to find a clear trend in favor of BPL or OSEM. By reviewing our set of images, we realized that images who gave the worst results were lesions near the mediastinum, unclearly visible without CT-fused scan and which can be mistaken with blood pool or muscle signal. All lesions located in the center of the lung with well-defined edges showed a similar or better detectability with BPL compared to OSEM.

Our detectability and quality evaluation has nevertheless strong limitations, as we decided to evaluate the randomized subset of images in one session, with no waiting time that could help the observers to memorize the location (20 lesions on FMISO or FAZA for 76 images). Moreover, we chose to only show one 2D image for each lesion, instead of a 3D scan to reduce the analyzing time and obtain the participation of more physicians. We also chose to not collect the false-positive findings as all physicians were aware of the presence a unique lesion on each 2D slice and since our aim was only to evaluate the potential gain of detectability achieved with BPL compared to OSEM.

Regarding the image quality analyses, scores were higher in 28% of cases using BPL while 27% of cases were in favor of OSEM. There was no change of quality score in 45% of cases. There were more cases of images classified as “good” or “excellent” in BPL than OSEM but also more cases of “uninterpretable”. As for detectability, the images that gave the worst quality were lesions unclearly visible, near mediastinum, or muscle with a low signal. Finally, the lesions located in the center of the lung presented identical or better-quality score in BPL compared to OSEM. An interesting part of our study is that our set of images was evaluated by nuclear physicians from another facility, who use BPL daily, unlike ours, and could point out a center effect bias. Based on our result, there did not seem to be a major difference in detectability nor quality between physicians from the 2 facilities.

We chose to realize this evaluation only for one *β* value of 350 determined with the phantom analysis instead of using more values for the clinical evaluation. The main reason is that we wanted as many physicians as possible to take part of this evaluation, as we did not know if there would be a lot of variability on detection and quality appreciation since there was no study realized about this subject in the literature. While our results suggest that the detectability is not modified by the reconstruction method, the quality evaluation seems to be way more observer dependent as some physicians prefer smoother images and others prefer sharper and more contrasted images (but also noisier).

Another limitation of our study concerns the use of spheres in the phantom of much smaller sizes than the lesions found in our patients. The real issue of detectability arises for small lesions. Since the aim of this work was to evaluate the interest of BPL on small lesions, we found it more interesting to focus on micro-spheres in our phantom study. Unfortunately, the patients’ lesions were finally larger than those used in the phantom, which could reduce the clinical relevance of our work. Nevertheless, as explained previously, results from Caribé et al. [12] using bigger spheres are in line with our results regarding the optimal beta reconstruction parameter.

Our study is limited by the small number of patients included and must be updated with the inclusion of other patients.

To our knowledge, this is the first study that evaluates BPL in hypoxia PET/CT and can play an important role in further studies about radiotherapy segmentation of hypoxic volumes.

## Conclusion

Our phantom study showed a better CRC vs noise trade-off for Q.Clear with a *β*350 compared to OSEM. While our phantom and clinical analysis for BPL realized with a beta value of 350 showed a significant increase in quantitative parameters and lesion contrast, we did not observe any significant changes in lesion detectability or image quality in comparison to OSEM. The variation in hypoxic volume by BPL depends on the method used but the SUV > 1.4 thresholding method seems to be the more robust and was not impacted by the reconstruction method (BPL or OSEM).

## Data Availability

The datasets generated during and/or analyzed during the current study are not publicly available but are available from the corresponding author on reasonable request.
